# A look in the mirror - body exposure in clinical practice

**DOI:** 10.1186/s40337-025-01262-6

**Published:** 2025-04-18

**Authors:** Jessica Werthmann, Eva Naumann, Silja Vocks, Jennifer Svaldi, Andrea S. Hartmann

**Affiliations:** 1 Institute of Psychology, Department of Clinical Psychology and Psychotherapy, Albert-Ludwigs-University Freiburg, Freiburg, Germany; 2 Institute of Psychology, Department of Clinical Psychology and Psychotherapy, Eberhard Karls University Tübingen, Tübingen, Germany; 3 Institute of Psychology, Department of Clinical Psychology and Psychotherapy, University of Osnabrück, Osnabrück, Germany; 4 Department of Psychology, Unit of Clinical Psychology and Psychotherapy of Childhood and Adolescence, University of Konstanz, Konstanz, Germany

**Keywords:** Body exposure, Exposure therapy, Cognitive-Behavioural therapy, Eating disorders, Dissemination, Evidence-based practice, Clinician anxiety, Therapist drift, Therapist’s characteristics, Research-Practice gap

## Abstract

**Background:**

The dissemination of evidence-based techniques is critical for the successful treatment of eating disorders in clinical practice. A growing number of studies suggests that body exposure is an effective technique to treat body image disturbance in eating disorders. However, the dissemination of body exposure among psychotherapists in clinical practice remains unclear.

**Methods:**

An online survey was conducted among licensed psychotherapists in Germany. The dissemination of body exposure in clinical practice, psychotherapists’ characteristics (such as clinical training, attitudes towards exposure, confidence), and therapists’ experiences with benefits and side-effects of body exposure were assessed.

**Results:**

Data of 230 psychotherapists were analysed. More than half of them (58.3%) applied body exposure in their clinical practice. Yet, body exposure was only offered to 37.3% of their eating disorder patients. Moreover, 56.7% of psychotherapists delivering body exposure indicated that they have not received any training in this technique. Self-reported confidence in delivering body exposure correlated significantly with the number of patients treated with body exposure. Psychotherapists who applied body exposure reported minor side-effects and that the majority of their patients profited from this technique.

**Conclusions:**

Our results offer insights into the dissemination of body exposure in clinical practice in Germany. Overall, body exposure is still underused considering the empirical evidence demonstrating the potential of body exposure to treat body image disturbances effectively. Moreover, with regard to potential barriers of using body exposure, our data suggest that training opportunities for clinicians may facilitate the dissemination of this technique in clinical practice.

**Supplementary Information:**

The online version contains supplementary material available at 10.1186/s40337-025-01262-6.

## Abstract (fr)

Body exposure is an evidence-based technique to treat body image disturbances. In this study, we assessed the use of body exposure among psychotherapists in clinical practice in Germany and found that body exposure is still underused in the treatment of eating disorders. Our data call for more training opportunities in this area, particularly as confidence in this technique is associated with more application.

Body image disturbance is a core symptom of eating disorder (ED) pathology (American Psychiatric Association 2013; Fairburn et al. 2003; Stice and Shaw 2002). Residing body dissatisfaction contributes to relapse after treatment (Jacobi et al. 2004). Targeting body dissatisfaction is therefore an integral part of ED treatment in several treatment manuals (e.g., Legenbauer and Vocks 2014; Svaldi and Tuschen-Caffier 2018; Tuschen-Caffier and Florin 2012; Vocks et al. 2018). A meta-analysis on the effects of interventions for body dissatisfaction demonstrated that body exposure (BE) is a promising technique towards this aim (Alleva et al. 2015). In brief, BE entails the repetitive and systematic description of oneself, for example in the mirror, usually under therapeutic guidance. This technique can be used for adult and adolescent patients with body dissatisfaction, however research testing BE in adolescents is scarce (Biney et al. 2021; Hartmann et al. 2021).

Similar to exposure in anxiety disorders, different concepts underlying its efficacy have been investigated. Most prominently, these focus on habituation (Díaz-Ferrer et al. 2015; Hilbert and Tuschen-Caffier 2004; Moreno-Domínguez et al. 2012; Trentowska et al. 2013, 2017; Vocks et al. 2007b, 2008). Yet, recently alternative theoretical concepts, including attention bias modification (e.g., Glashouwer et al. 2016; Krohmer et al. 2022), cognitive change (e.g., Baur et al. 2022) and reduction of body perception distortion (e.g., Lewer et al. 2017) have been tested as well (see for an overview e.g., Hartmann et al. 2021).

BE has positive effects on several ED-related symptoms such as body dissatisfaction, negative mood, low self-esteem and dysfunctional body-related information processing (Griffen et al. 2018; Hartmann et al. 2021; Krohmer et al. 2022). However, despite its efficacy, very little is known about the use of BE in clinical practice. Notably, against recommendations in clinical guidelines for a range of mental disorders (e.g., Arbeitsgemeinschaft der Wissenschaftlichen Medizinischen Fachgesellschaften 2019; National Institute for Health and Care Excellence, 2017), exposure is generally often poorly disseminated (Becker-Haimes et al. 2017; Cook et al. 2004; Kline et al. 2021). For example, 87% of more than 2000 participating mental health clinicians in the U.S. indicated that they have *never* applied exposure-based techniques in their daily practice (Cook et al. 2010). Similarly, a survey of licensed psychotherapists in Germany found that less than half of the respondents used exposure when treating patients with anxiety disorders (Pittig and Hoyer 2017).

This striking gap between evidence and practice has stimulated considerable research, particularly in the field of anxiety disorders. Several factors have been identified and associated with the limited use of exposure. Most importantly, negative attitudes towards exposure and exposure-related concerns (e.g., emotional distress or the expectation that patients may not tolerate exposure) reduce the likelihood of delivering exposure in daily practice for anxiety disorders (Becker-Haimes et al. 2017; de Jong et al. 2020; Deacon et al. 2013; Kline et al. 2021; Schumacher et al. 2019). Other potentially related factors include therapist characteristics (e.g., age and experience (de Jong et al. 2020; Deacon et al. 2013; Finch et al. 2020), clinical setting (de Jong et al. 2020; Harned et al. 2013; Kline et al. 2021; Milgram et al. 2022; Pittig and Hoyer 2017), as well as therapists’ misunderstanding of the exposure rationale (Kline et al. 2021). However, emerging research suggests that training opportunities in exposure may facilitate the implementation of exposure in daily practice by influencing attitudes, concerns and confidence regarding the delivery of exposure (Farrell et al. 2016; Trivasse et al. 2020; Wright and Waller 2020).

With regard to the implementation of BE in the treatment of EDs, there is a lack of evidence about the dissemination in daily practice and factors that may influence use of BE. Against this backdrop, the primary aim of this study was to assess the prevalence of applying BE among licensed psychotherapists treating patients with EDs in clinical practice in Germany. A secondary aim was to assess how psychotherapists’ factors such as age, gender, clinical background, negative beliefs and attitudes towards BE are associated with the delivery of BE in ED treatments. Finally, we wanted to evaluate psychotherapists’ experiences with the benefits and side-effects of BE in clinical practice.

## Method

### Recruitment

Participants were included if they indicated to hold a formal license as psychotherapist in Germany (i.e., “Approbation”), including adult psychotherapists, child and adolescent psychotherapists as well as medical doctors with psychotherapy training. We recruited licensed psychotherapists via their registered email at the Association of German Health Insurance Providers (i.e., the “Kassenärztliche Vereinigung”), in which all licensed psychotherapists must register. We contacted psychotherapists in several federal states of Germany (i.e., Baden-Wurttemberg, a section of North Rhine-Westphalia, Rhineland Palatinate, Brandenburg, Saxony and Schleswig-Holstein). Additionally, inpatient clinics in these federal states were hand-searched via internet and the head psychologist or the director of the clinic was contacted via email with the request to pass on the study invitation to psychotherapy-licensed employees.

This invitation email encouraged licensed psychotherapists to participate in an online survey about their experiences with BE in the treatment of EDs. The email contained a link to our electronic survey and participants were informed that they would be offered a free online workshop on BE if they completed the survey.

### Materials

The online survey was programmed and distributed via the German online survey platform Unipark (Tivian XI GmbH, 2021) and consisted of 25 main questions which contained, partly, further sub-questions, see supplementary materials for an overview and Open Science Framework Project (https://osf.io/fz9ys/) for the complete German-language survey.

Overall, the survey contained questions about the dissemination of BE in daily practice, (e.g., if therapists have used BE and whether other techniques have been used to treat body image disturbances; aim 1: dissemination). It also contained questions concerning the psychotherapist’s characteristics (e.g., demographics, clinical background, confidence with BE, positive attitudes and negative beliefs; aim 2: therapist factors associated with the use of BE) and, finally, there were also questions concerning potential benefits and side-effects of BE (aim 3: experiences in clinical practice). Most questions were measured with a four-point Likert scale (ranging from 1 “Not applicable at all” to 4 “Very much applicable”). Some questions required single answer selection (e.g., “Did you ever apply body exposure?” “Yes”/ “No”). Moreover, a few open answer questions (e.g., “Do you have any further comments on the survey”) were included; see supplementary materials and Open Science Framework Project for more details. Additionally, we also included several questions about the specific implementation of BE (e.g., duration of BE sessions, use of homework), which are part of a different research question.

### Procedure

The survey was online between November 2019 and August 2022. Upon accessing the survey, a brief definition of BE, study aims and eligibility criteria as well as a policy statement on confidentiality and data security were provided. Participants were informed that all responses were assessed, analysed, and stored anonymously and were then given the choice to provide informed consent online. Participants could access the survey only after they had provided their informed consent. As compensation for their time and effort, we offered an online BE workshop to the participants. Interested participants had then to indicate their email address in a separate survey accessible over a link in order to receive access to the workshop invitation. This separate link guaranteed the anonymous data collection of responses during the survey. The study protocol and survey questions were approved by the Ethical Committee of the Albert-Ludwigs University Freiburg, Germany (494/19).

### Data analyses

Data were analysed with the statistical software program SPSS. If not indicated otherwise, mean values and standard deviations of Likert-scale items were calculated. Two questions about attitudes towards exposure in general and two questions about attitudes towards BE specifically were asked, using similar wording (“In general, I believe that (body) exposure is an important technique” and “I use (body) exposure regularly”). These questions were designed based on prior research by Becker et al. (2004), see supplementary materials for more details. A compound score of these four questions was computed comprising positive attitudes towards (body) exposure.

Negative beliefs towards body exposure were evaluated with 13 questions adapted from the German version of the therapist beliefs about exposure scale (TBES; Deacon et al. 2013; Schumacher et al. 2019) and a sum score was computed, see supplementary materials for more details. Missing values of these items were not imputed and cases with missing values were excluded from analyses on negative beliefs. Confidence of BE was measured based on the mean of two questions about knowledge and self-efficacy when using BE, see supplementary materials for more details.

When applicable (e.g., when comparing practitioners using BE with those who do not use BE) independent sample *t*-tests were used and degrees of freedom were adjusted if variances were unequal and are reported accordingly. When correlations of two categorical nominal variables were conducted (e.g., gender and use of body exposure), cross-tabulation correlations were conducted and X^2^ (Chi-Square) statistics were reported, otherwise Pearson’s correlation coefficient was reported.

## Results

Due to the unknown number of people who received the invitation email, it was not possible to calculate a precise response rate. Overall, 1.132 people accessed the survey link and 668 people (59%) started the survey, but discontinued after the first page which stated the purpose of the survey. Overall, 283 people (25%) completed the survey. Of the 283 participants who completed the survey, 21 were not licensed psychotherapists and 26 indicated they were medical doctors without formal psychotherapy training, therefore these participants (*n* = 47) were excluded from further analyses. Of the remaining participants (*n* = 236), five therapists indicated that they were trained in other therapy approaches than cognitive behavioural therapy and psychodynamic therapies. As these numbers were too small for meaningful separate analyses (e.g., concerning the association with usage of BE), we chose to exclude those five therapists from further analyses. One participant who indicated to use BE was excluded, as their description of the applied BE procedure was not in concordance with usual procedures. Overall, this resulted in a final sample of 230 licensed psychotherapists for the analyses.

A total of 155 of the eligible 230 participants were psychotherapists for adults (67.4%), 56 worked as child - and adolescent psychotherapists (24.4%) (10 of those worked as child and adolescents as well as adult psychotherapists) and 19 worked as medical doctor with psychotherapy training (8.3%). The majority of participating psychotherapists were cognitive behavioural therapists (78.3%) and 21.7% were psychodynamic psychotherapists. Most participating psychotherapists were women (82.6%; 0.4% specified as non-binary) and mean age was 45.3 years (*SD* = 9.4, *Range* = 24–72), see also Table 1 for sample characteristics.

**Table 1:** Characteristics of participating psychotherapists

Characteristics		Overall sample (*N* = 230)	BE-therapists (*n* = 134)	Non-BE therapists (*n* = 96)	Statistics^g^
Age (*M*,* SD*)		45.31 (9.43)^a^	45.18 (9.13)	45.50 (9.88)	*t*(227) = 0.25
Gender (f/m/non-binary)		190/35/4^a^	111/19/3^b^	79/16/1	*Χ*^*2*^*(df = 3*, *N = 230) =* 1.41
Psychotherapy Training					*Χ*^*2*^*(df = 1*,* N = 230) =* 27.34^**^
	CBT	180	121	59	
	PD	50	13	37	
Specialisation					*Χ*^*2*^*(df = 2*,* N = 230) =* 4.20
	Psychological Psychotherapist for Children & Adolescents	56	32	24	
	Psychological Psychotherapist for Adults	155	95	32	
	Medical doctor with psychotherapy training	19	7	12	
Years of Experience (*M*,* SD*)		11.77 (7.70)	12.51 (7.32)	10.74 (8.13)	*t*(228) = 1.72
% of ED patients (*M*,* SD*)		12.88 (12.23)	14.26 (13.13)	10.95 (10.62)	*t* (224.6) = 2.11^*^
Negative beliefs^c^ (*M*, *SD*)		25.18 (5.26)	23.96 (4.57)	26.95 (5.70)	*t*(220) ^d^ = 4.32^**^
Positive attitudes^e^ (*M*, *SD*)		12.21 (2.59)	13.10 (2.32)	10.97 (2.43)	*t*(228) = 6.74^**^
Confidence^f^ (*M*, *SD*)		2.05 (0.80)	2.44 (0.72)	1.51 (0.55)	*t*(227.1) = 11.08^**^

Note. *M* = Mean, *SD* = Standard deviation, f = female, m = male, BE therapists = therapists who indicated to have applied BE; non-BE therapists = therapists who reported to have not applied BE. ^a^ based on *n* = 229, because one answer was missing. ^b^ based on *n* = 133, because one answer was missing. ^c^ based on a sum score comprising 13 questions of the BE-adapted German Version of the Therapist Beliefs Scale (Deacon et al. 2013; German Version see Schumacher et al. 2019) range: 13–52, higher scores = more negative beliefs, ^d^ based on *n* = 222, because eight participants had missing values. ^e^ based on the sum score comprising four statements on the use of BE (two questions) and exposure in general (two questions), range 4–16, higher scores = more positive attitudes. ^f^ based on the average score of two questions on knowledge and security when using BE (range 1–4, higher scores = more confidence).^g^ Statistics refer to a comparison between BE and non-BE therapists, the statistical test applied is indicated in the respective cell. ^*^ Significant at *p* <.05, ^**^ significant at *p* <.001

On average, participating clinicians reported a working experience of 11.8 years (*SD* = 7.7, *Range* = 0–45) with EDs and that patients with EDs made up about 12.9% (*SD* = 12.2, *Range* = 0–80%) of their total patient load. Most clinicians indicated that they had seen about one to five patients of each ED diagnosis category: Anorexia Nervosa (AN; 48.7% of therapists), Bulimia Nervosa (BN; 45.7% of therapists), Binge Eating Disorder (BED; 38.7% of therapists) and Other Specified Feeding and Eating Disorders (OSFED; 40.9% of therapists). A minority of therapists indicated that they treated more than 20 patients with AN (11.7%), BN (11.7%), BED (10.0%) and OSFED (13.0%). On average, more than half (53.7%) of participating therapists indicated that they treated their ED patients in an outpatient (vs. inpatient) and individual (vs. group format) (65.5%) client setting.

### Dissemination of body exposure in clinical practice

The majority of participants indicated that they have used BE in clinical practice (58.3%). Therapists who used BE delivered this technique, on average, to 12.4 patients (*SD* = 17.0, *Range* = 0–100) and treated 37.3% of their total ED patient load with BE.

The majority of therapists who used BE delivered BE specifically to patients with EDs (93.3%), particularly to patients with AN (75.4% indicated use of BE for AN) and BN (56.7% indicated use of BE for BN). BE was less often applied in the treatment of OSFED (40.3% indicated use of BE for OSFED) and treatment of BED (20.9% therapists indicated use of BE for BED).

Additionally, 35.1% of therapists using BE indicated that they delivered BE to patients with Body Dysmorphic Disorder (BDD) and 21.6% used BE for patients with other psychological or medical problems (e.g., people with lower self-esteem, people with obesity, people with posttraumatic anxiety disorder and body dysphoria in transgender individuals).

The majority of therapists using BE (87.3%) indicated that they have also used other techniques to treat body image disturbances, such as cognitive interventions (81.3%), drawing of the body silhouette (59.0%) and estimating body size with a rope/tape measure (32.8%). Additionally, 15.7% reported to apply additional techniques such as mindfulness, body-related imagery exercises and body-related chair dialogues. Therapists who applied BE were also asked to indicate the percentage of using BE in proportion to all applied techniques to treat body image disturbances. Results yielded that BE made up, on average, 24.5% of all techniques used to treat body image disturbances (*SD* = 21.0; *Range* = 0–100). However, of note, 38.5% of therapists who did not use BE indicated that they do not apply any other techniques to treat body image disturbances. Thus, the answering pattern of this group of participants suggests that no specific treatment for body image disturbances is implemented during treatment.

### Associations of psychotherapists’ characteristics, attitudes and beliefs with the dissemination of body exposure

To evaluate if and how specific characteristics of clinicians relate to the dissemination of BE, we conducted correlational analyses of clinicians’ demographic variables, experience-related variables (such as proportion of ED patients and type of psychotherapy training) and attitudes with the delivery of BE.

Age (*r*(229) = − 0.02, *p* =.80) and gender (X^*2*^(*df* = 3, *N* = 230) = 1.41, *p* =.70) were not significantly associated with the use of BE (i.e., if BE was used: yes/no). Duration of working experience (in years) with EDs (*r*(230) = − 0.11, *p* =.09) and professional specialisation (i.e., adult, child - and adolescents, or medical doctors with psychotherapy training (X^*2*^(*df* = 2, *N* = 230) = 4.20, *p* =.12) yielded non-significant associations with the application of BE. Instead, a post-hoc correlation analysis yielded that being trained in BE was significantly associated with implementation of BE, X^*2*^(*df* = 1, *N* = 230) = 25.03, *p* <.001: the majority of therapists who indicated that they were trained in BE also indicated that they implement BE in daily practice (82.5%). In contrast, half of therapists, who did not receive a training for BE (52.5%) indicated that they have never implemented BE in daily practice.

The use of BE also differed significantly depending on the type of psychotherapy training (i.e., cognitive-behavioural therapy, psychodynamic therapies): 67.2% of participating cognitive-behavioural therapists delivered BE, whereas 26% of psychodynamic therapists indicated to use this technique, X^*2*^(*df* = 1, *N* = 230) = 27.34, *p* <.001. Moreover, compared to therapists who never used BE, therapists using BE treated a significantly larger proportion of patients with EDs (14.3% vs. 10.9%) within their total patient load, see Table 1. Similarly, a significant correlation (*r*(230) = 0.13, *p* =.04) demonstrated the positive association of BE application and proportion of ED patients treated within total patient load.

Moreover, therapists who never used BE reported overall significantly more negative beliefs about (body) exposure and expressed less positive attitudes towards (body) exposure than therapists using BE. In addition, therapists who applied BE were significantly more confident in BE, see Table 1. Training in BE was also significantly associated with positive attitudes towards (body) exposure and confidence in BE, (*r*(230) = 0.25, *p* <.001 and *r*(230) = 0.40, *p* <.001, respectively). Training in BE was, however, not associated with negative beliefs about (body) exposure (*r*(230) = − 0.05, *p* =.46).

Within the group of therapists who applied BE, confidence in BE correlated significantly positively with the number of patients treated with BE, *r*(134) = 0.51, *p* <.001 and the percentage of using BE in proportion to other techniques used to treat body image disturbances, *r*(134) = 0.46, *p* <.001. Results yielded that positive attitudes towards (body) exposure (based on the compound score) correlated positively with the proportion of patients treated with BE, *r*(134) = 0.27, *p* =.002. Negative beliefs about BE correlated negatively with the proportion of using BE as a technique to treat body image disturbances, *r*(131) = − 0.28, *p* =.001. Within this group of therapists, negative beliefs about BE did not correlate significantly with age, years of working experience, or with the percentage of patients with eating disorder treated, *r*(131) = 0.12, *p* =.19, *r*(131) = − 0.12, *p* =.18 and *r*(131) = − 0.12, *p* =.18, respectively.

### Evaluation of benefits and side-effects of body exposure in daily practice

Therapists using BE reported that, on average, the majority (62.1%) of their patients profit from BE. A range of several potential benefits and side-effects of BE were then rated on 4-point Likert scales (answering options were: “not applicable at all” (1), “not applicable” (2), “applicable” (3), “totally applicable” (4), see Open Science Framework (https://osf.io/fz9ys/) for the complete questionnaire and all answering options). Overall, therapists who applied BE rated an improved regulation of negative body-related emotions (*M* = 3.43, *SD* = 0.6), a reduction of biased body-related perception (*M* = 3.40, *SD* = 0.6) as well as a reduction in negative body-related cognitions (*M* = 3.40, *SD* = 0.6) as most important indicators of BE benefits. Moreover, within the group of therapists who implemented BE, confidence correlated significantly with the rate of patients who they reported improved, *r*(131) = − 0.12, *p* =.18. In other words, based on these therapists’ self-reports, BE was more effective when the therapist had a high confidence in BE *r*(134) = 0.37, *p* <.001.

As negative side effects, therapists who applied BE observed most often self-depreciation (*M* = 2.8, *SD* = 0.8) and an increased focus on negative cognitions and emotions (*M* = 2.4, *SD* = 0.8). Drop-out from therapy was rated as least often experienced side effect with 98.5% of therapists who applied BE indicated that they have never or rarely experienced drop-out from therapy due to BE. Only two therapists (out of 134 therapists who applied BE, i.e., 1.4%) indicated that they have regularly (*n* = 1) or often (*n* = 1) experienced drop-out due to BE. No other serious side effects were reported in a voluntary open answer format by therapists.

## Discussion

The primary aim of this study was to quantify the dissemination of BE as an evidence-based technique for body image disturbances in patients with EDs in daily practice in Germany. In addition, we wanted to test whether therapist characteristics such as demographic background, clinical specialisation and attitudes towards exposure were associated with the uptake of BE. Moreover, we aimed to assess therapists’ perception of the benefits and side-effects of BE in clinical practice.

Most importantly, the results of our online survey suggest that BE is relatively well disseminated in clinical practice in Germany: More than half of all participating therapists (58%) indicated that they have used this technique. The percentage of therapists offering BE is relatively high, compared to the prevalence of other (disorder-specific) exposure techniques offered in clinical practice. For example, studies on the dissemination of exposure in treatment of anxiety disorders often reported the use of (specific) exposure techniques in less than half of their samples (e.g., Becker-Haimes et al. 2017; Freiheit et al. 2004; Moses et al. 2021; Pittig and Hoyer 2017). Perhaps therapists treating EDs tend to specialise in ED-specific treatment techniques such as BE because EDs are challenging to treat (Schmidt et al. 2016). This is in line with our data showing that about 43% of therapists who used BE received a specific BE training (which is also a fairly high percentage compared to similar research, see e.g., Becker et al. 2004). Likewise, about 71% of participating therapists (203 out of 283) responded to our offer of a free online BE-training as compensation for their effort to complete the survey.

Although the majority of therapists reported using BE, it is important to note that BE was only delivered to about a third of all patients (i.e., 37%). This finding suggests that there is room for improvement in the dissemination of BE, especially considering that BE has clearly acquired the strongest evidence base as a treatment technique for body image disturbances at this point (Alleva et al. 2015; Hartmann et al. 2021; Naumann et al. 2022). Although 87.3% of therapists using BE reported also using additional techniques (mainly cognitive interventions) to treat body image disturbances, 38.5% of therapists who did not use BE reported also not using *any* additional technique targeted at body image disturbances. This finding is concerning given that body image disturbances are a core symptom of EDs and pose a risk of relapse if left untreated (Glashouwer et al. 2019; Jacobi et al. 2004).

Although the survey-completion rate of 25% was similar to previous research on exposure provision in clinical practice in Germany (e.g., 28.6% in Pittig and Hoyer 2017; 25.5% in Schumacher et al. 2019), one explanation for the comparably high percentage of BE dissemination may be that a selection bias occurred in the current study. During recruitment, we took active steps to avoid selection bias (e.g., by emailing all registered therapists and clinics, regardless of specialisation and professional background, and by emphasizing that therapists were invited to participate regardless of their experience with BE). However, it is possible that therapists who were familiar with the delivery of BE were more interested in supporting this research and therefore completed the survey, leading to an overestimation of the prevalence of usage of BE in clinical practice. The results should therefore be considered with this potential limitation in mind. Furthermore, the offer of a free online BE workshop as incentive for participation may also have contributed to a selection bias of therapists who were interested in BE. Future studies may consider other incentive methods (e.g., monetary compensation) to reduce this potential bias.

With regard to our secondary aim (i.e., the association between therapist characteristics and the use of BE), we found that the use of BE was more common among cognitive behavioural therapists than psychodynamic therapists– a finding that may in itself be explained by the different underlying theoretical conceptions of the therapeutic process. This is consistent with previous research showing that clinical orientation determines fundamental differences in ideas about treatment approaches (i.e., symptom-orientated cognitive behavioural approach vs. psychodynamic approaches see von Ranson and Robinson, 2006). Several previous (but not all) studies indicated that exposure is more often used by cognitive behavioural therapists than by therapists of other clinical orientations (e.g., de Jong et al. 2020; Langthorne et al. 2023).

Notably, our results indicated that therapists who never applied BE endorsed more negative beliefs about BE and held less positive attitudes towards exposure in general and BE in particular. This is consistent with numerous studies showing that negative beliefs about exposure hinder the implementation of exposure in clinical practice, despite overwhelming empirical evidence to the contrary (de Jong et al. 2020; Deacon et al. 2013; Langthorne et al. 2023). In contrast, positive attitudes towards BE and self-reported confidence in delivering BE were significantly associated with higher use of this therapeutic technique. These findings highlight the potential link between positive attitudes and the use of BE. These results also suggest that increasing confidence in BE delivery may be associated with greater use which was also found in a study on exposure training for therapists (Harned et al. 2013). Note, however, that additional interventions may be needed to increase the implementation of exposure techniques in daily practice, for example interventions using implementation intentions or interventions reducing concerns and anxiety about exposure use (Farrell et al. 2016; Schumacher et al. 2015; Trivasse et al. 2020).

In the current sample, therapists’ age and gender did not correlate with the use of BE. This is in contrast to the results of previous research which found that female clinicians and older clinicians were less likely to use exposure in their daily practice (de Jong et al. 2020; Deacon et al. 2013). A possible explanation for this divergent finding may be that all participating therapists were licensed psychotherapists, as per the inclusion criterion, who may hold less negative beliefs about exposure in general and may therefore may be more likely to use exposure (Becker-Haimes et al. 2017; Deacon et al. 2013; Kline et al. 2021). This is in line with reports of another study among German therapists, which observed fewer reservations about exposure than generally found in comparable studies in the US (Schumacher et al. 2019). Notably, cognitive behavioural psychotherapy training in Germany often includes many opportunities to learn and to train exposure, which may therefore lead to fewer reservations about exposure and therefore to a higher overall delivery rate. Another explanation could be that BE is more commonly delivered by female therapists, as the majority of patients with EDs are female and prefer female therapists (Vocks et al. 2007). This may have affected our data, as the majority of participating therapists were female.

In terms of therapists’ perceptions of the benefits and side-effects of BE, our results showed that BE was overall perceived to be effective: therapists using BE reported positive effects of BE for > 60% of their patients. Moreover, confidence in BE correlated significantly positively with the percentage of patients who improved through BE. Thus, therapists who were more confident in BE, indicated that a higher number of their patients profited from BE. This result may indicate that boosting confidence of therapists in BE delivery, for example through training, may amplify treatment effects. This interpretation is in line with previous research showing a positive effect of therapists’ confidence and competence on treatment outcome and treatment motivation (Bartle-Haring et al. 2022; Seewald and Rief 2024).

Therapists who applied BE described the benefits of BE as the regulation of body-related negative emotions and cognitions as well as the reduction of distorted body image as equally important determinants of treatment success. This observation by therapists converges with research showing significant improvements in negative body-related emotions and biased attentional processing of body parts in response to BE (Griffen et al. 2018; Hartmann et al. 2021; Krohmer et al. 2022; Naumann et al. 2022). Thus, the benefits of BE in controlled settings appear to be consistent with what is observed in clinical practice. Note, however, that this data is based on therapists’ evaluation of benefits and not on clients’ self-report in the current study. Moreover, we did not assess how therapists came to this evaluation (e.g., based on clinical impression, patient self-report or questionnaires).

Furthermore, in the current study, therapists who applied BE, indicated that negative side-effects were rarely experienced in clinical practice. This is particularly relevant as a previous review of the adverse effects of BE highlighted the inconclusive evidence on the adverse effects of BE (Griffen et al. 2018). Moreover, this finding may also indicate that clinicians’ fears of imposing a heavy emotional distress on patients when using exposure-based techniques, such as BE, are not consistent with clinical experience in daily practice as reported by participants in this study. Instead, this finding should encourage clinicians to use BE despite their potential inhibiting fear of not being sure about the outcome of a BE session (see for a similar argumentation Mulkens et al. 2018). Using BE as often as possible will increase confidence in this technique, which will in turn increase the use of BE, as our results suggest. Training in BE and supervision may further facilitate this effect (Michael et al. 2021; Moses et al. 2023).

Overall, we conclude that even though the majority of therapists in our sample were familiar with BE, BE is applied in less than half of all treated patients. This is particularly important against the background that BE may be currently the best technique available to treat body dissatisfaction (Alleva et al. 2015), at least within a cognitive behavioural therapy-treatment context, and a substantial number of patients may not receive any interventions for body image disturbances. Thus, these results call for continued development to foster optimal treatment of EDs in clinical practice.

One way to increase the uptake of BE within cognitive behavioural therapy treatment is through training and supervision opportunities. Our findings highlight the importance of training in BE with the uptake of BE, attitudes towards BE and confidence in BE. This is in line with previous research that emphasized the potential of addressing positive attitudes towards exposure directly during therapy training in order to increase the dissemination of exposure in clinical practice (Deacon et al. 2013; Farrell et al. 2016; Simmons et al. 2008; Trivasse et al. 2020). However, note, that in a survey among German therapists, more training and/or supervision opportunities were named as least important factors influencing the decision to apply exposure for patients with anxiety disorders (Pittig and Hoyer 2017). In that specific study, environment-related factors (such as uncertainty about remuneration and insurance) were named as most important for the (non)-utilization of exposure. Moreover, Schumacher and her colleagues (Schumacher et al. 2014, 2015) found that exposure was linked to high stress responses in clinicians during delivery, which may impact the likelihood and caution of exposure delivery (Deacon et al. 2013; Langthorne et al. 2023). Thus, it is important for future research to determine which specific factors can encourage and can motivate psychotherapists to implement BE to the majority of their patients with body image disturbances. Similarly, it is important to gain a better understanding of barriers that impede therapists from using BE.

In conclusion, our survey revealed that therapists working with ED perceive BE as a beneficial technique for addressing body image disturbances in clinical practice. However, the finding that BE is only applied in about a quarter of all ED cases, and that confidence and positive attitudes are significantly associated with the use of BE highlights the need to lower therapists’ individual barriers to use this disorder-specific exposure technique.

## Electronic supplementary material

Below is the link to the electronic supplementary material.

Supplementary Material 1

## Data Availability

The online survey (in German language) and the dataset with relevant variables analysed during the current study are available in the Open Science Framework repository, osf.io/fz9ys/.

## Abbreviations

AN: Anorexia Nervosa
BE: Body Exposure
BED: Binge Eating Disorder
BDD: Body Dysmorphic Disorder
BN: Bulimia Nervosa
ED: Eating Disorder
OSFED: Other specific feeding and eating disorders
