# Supplementary material for: A look in the mirror - body exposure in clinical practice
Source: J Eat Disord. 2025 Apr 18;13:69. doi: 10.1186/s40337-025-01262-6 (PMC12007345; doi:10.1186/s40337-025-01262-6)
Supplement: Supplementary file 1 — Supplementary Material 1 [file 40337_2025_1262_MOESM1_ESM.docx]

**Supplementary material S1: Overview of survey questions per study aim.**

We created a list of suitable questions based on comparable questionnaires used in previous research (e.g., Pittig & Hoyer, 2017; Schumacher et al., 2019) and based on suggestions of BE experts. In these supplementary materials, we provide an overview of the specific questions and their answering format per study aim as well as their operationalisation for analyses, if applicable.

*1. Questions regarding the dissemination of BE in clinical practice.*

To assess the dissemination of BE in clinical practice, participants had to indicate whether they applied BE to their patients (i.e., “Did you ever used BE as technique to treat body image disturbances” – Answering options were “Yes/No”). The survey also asked participants how many patients they have treated with BE (i.e., “Indicate, roughly, how many people you have treated with BE” and “Indicate the proportion of all patients with ED for whom you used BE in comparison to all treated patients with ED” – Answering options were an open format to enter numbers). Specifically, participants also indicated for which ED diagnoses they used BE (i.e., “Indicate the diagnosis of patients you have treated with BE” – Answering options were: “EDs”, “AN”, “BN”, “BED”, “atypical ED and OSFED”, “body-dysmorphic disorder”, “other disorders, namely …” [open answer option]). Moreover, we also assessed if other techniques were used to address body image disturbance (i.e., “Do you use other techniques to treat body image disturbances” – Answering options were: “No”/”Yes” – If yes, multiple choice options on specific other techniques followed: “Estimating body size with a rope”, “Drawing body silhouette”, ”Cognitive interventions”, “Others, namely…” [open answer option]). Participants also indicated the percentage of using BE in proportion to all techniques used to treat body image disturbance (i.e., “Indicate the percentage of using BE in proportion to other techniques to treat body image disturbances” – Answering option was an open format to enter a percentage number). Additionally, participants were asked whether they have received a training in BE (“Did you ever follow a formal training in BE?” – Answering options were “Yes/No”).

*2. Questions regarding psychotherapist factors potentially associated with the dissemination of BE*

Based on previous research, psychotherapists’ factors which may influence the dissemination of BE were assessed in the survey (de Jong et al., 2020; Deacon et al., 2013; Langthorne et al., 2023; Pittig & Hoyer, 2017; Schumacher et al., 2019). We assessed age and gender as demographic variables. Additionally, to evaluate the clinical background, psychotherapists were asked to provide information on their professional background (“What is your profession?” – Answering options were multiple choice options: “Psychological psychotherapists” (i.e. licenced psychotherapist specialised on working with adults), “Child-and adolescents psychotherapist” (i.e. licenced psychotherapists, specialised on working with children and adolescents) “Medical psychotherapists” (i.e., someone who studied medicine and then completed a psychotherapist training in Germany leading to a licence to practice psychotherapy as medical doctor), “Medical doctor” (i.e. someone who holds a licence as medical doctor, e.g. psychiatrists) and to report their clinical orientation (“Please indicate your primary therapy orientation” – – Answering options were multiple choice options: “Cognitive behavioural therapy”, “Psychoanalytic therapy”, “Systemic therapy”, “Other, namely… [open answer option]).

To assess clinical experience, participants indicated their working experience with EDs in years (“How many years have you been working with ED patients?” – Answering option was an open format to enter numbers). Participants were also asked to estimated the proportion of ED patients in relation of their total patient load (“How large is the proportion of patients with ED in comparison to your total patient load?”) and to specify how many patients they have treated per ED diagnosis (“Please indicate how many patients with the following diagnosis you have treated so far” – Answering options were: “None”, “1-5”, “5 -10”, “10-15”, “10 -20”, “< 20” for AN, BN; BED, OSFED diagnoses, respectively).

To assess beliefs about exposure, items were partly based on the German version of the therapist beliefs about exposure scale (TBES; Deacon et al., 2013; Schumacher et al., 2019). We used 11 questions of the TBES (Items 1,2,3,4,6,9,13,14,15,20,21) which were suitable in the context of body exposure and merely replaced the wording in terms of “exposure” with “body exposure” if necessary (e.g., TBES item 6 “Exposure cannot be individualised” became “*Body* exposure cannot be individualised”). Additionally we included two questions on negative beliefs about body exposure based on expert opinion (i.e., “Body exposure is not adequate for comorbidities” and “Body exposure is unethical”). Thus, in total 13 items assessed negative beliefs about BE. All items were rated on 4-point Likert scale ranging from 1 (“not applicable at all”) to 4 (“absolutely applicable”). For analyses purposes, a sumscore was computed. Missing values were not imputed and cases with missing values were excluded from analyses. Sum scores could range from 13 to 52 with higher scores indicating more negative beliefs about BE.

Additionally, we also assessed positive attitudes towards exposure in general and towards BE specifically with two identical formulated questions, respectively (“I think exposure is an important technique”, “I use exposure regularly” and “I think body exposure is an important technique”, “I use body exposure regularly”). These questions were based on similar questions used by Becker and colleagues (Becker et al., 2004). Items were rated on a 4-point Likert scale ranging from 1 (“not applicable at all”) to 4 (“absolutely applicable”). Again, for analyses purposes, a sum score of these four items was created, comprising positive attitudes towards exposure and BE. Sum scores could range from four to 16 with higher scores indicating more positive attitudes towards exposure in general and body exposure specifically.

Confidence in applying body exposure with assessed with two questions (“I think I have a good expertise in body exposure” and “I feel confident when delivering body exposure”). Answers were rated on a 4-point Likert scale ranging from 1 (“not applicable at all”) to 4 (“absolutely applicable”). A mean value was calculated based on answering scores of these two question to analyse self-reported confidence in body exposure.

*3. Questions regarding benefits and side-effects of BE in clinical practice.*

To assess potential benefits of BE, we asked psychotherapists to estimate the percentage of patients who benefitted from BE (“Indicate the percentage of patients who profited from BE of all patients with whom you used BE” - – Answering option was an open format to enter numbers). To get a more specific idea of what benefits psychotherapists experienced, participants also rated indicators of benefits (“What would be the best indicator of benefits due to BE”) based on a list of eight items derived from previous research reporting benefits of BE (e.g., “Description of body becomes more positive”) and one optional free-answering format (“Other, namely…[open answer option]”). Ratings for each of these items were provided on a 4-point Likert scale ranging from 1 (“not applicable at all”) to 4 (“absolutely applicable”). Similarly, side-effects were assessed by asking to rate potential side-effects based on a list of six items derived from previous research reporting side-effects of BE or anecdotal reports by ED experts (e.g., “Drop-out of therapy”) and one optional free-answering format (“Other, namely…[open answer option]”). Again, ratings for each of these items were provided on a 4-point Likert scale ranging from 1 (“not applicable at all”) to 4 (“absolutely applicable”).
